# Efficacy and tolerability of Etravirine in HIV-1 adult patients: Results of a large French prospective cohort

**DOI:** 10.7448/IAS.17.4.19780

**Published:** 2014-11-02

**Authors:** Clotilde Allavena, Christine Katlama, Laurent Cotte, Pierre-Marie Roger, Pierre Delobel, Antoine Cheret, Claudine Duvivier, Isabelle Poizot-Martin, Bruno Hoen, Andre Cabié, Arnaud Cheret, Rima Lahoulou, Francois Raffi, Pascal Pugliese

**Affiliations:** 1 Infectious Diseases, CHU Hôtel Dieu, Nantes, France; 2 Infectious Diseases, AP-HP Hôpital Pitié Salpétrière, Paris, France; 3 Infectious Diseases, Hospices Civils de Lyon, Lyon, France; 4 Infectious Diseases, CHU Archet – Universite Nice Sophia Antipolis, Nice, France; 5 Infectious Diseases, CHU de Purpan, Toulouse, France; 6 Infectious Diseases, CHU Druon, Tourcoing, France; 7 Infectious Diseases, IHU Imagine, Necker Hospital – Descartes University, Paris, France; 8 Infectious Diseases, AP-HM Hôpital Sainte Marguerite, Marseille, France; 9 Infectious Diseases, CHU Besançon, Besançon, France; 10 Infectious Diseases, CHU Fort de France, Fort de France, France; 11 Janssen, Issy Les Moulineaux, France

## Abstract

**Background:**

IEtravirine (ETR) was approved in France in Sept 2008, to be used in combination with a ritonavir-boosted protease inhibitor (bPI) and others antiretrovirals (ARV) in HIV-infected pre-treated patients.

**Objectives:**

To describe in a real life setting efficacy and tolerability of ETR-including regimen and factors associated with virologic response.

**Method:**

In the French DatAIDS cohort including 18,647 patients, we selected patients who initiated an ETR-including regimen between September 2008 and July 2013. Demographic data and clinico-biological data were collected from the standardized electronic medical record Nadis^®^. Analyses were done in patients starting ETR and sub-analyses were performed in pre-treated patients starting ETR for virologic failure (VF) or maintenance (MT) therapy, with or without bPI.

**Results:**

2083 patients (ARV-naïve n=77, VF n=1014, MT n=992) were included: median age 47 years, 73.3% male, median duration of HIV infection 15.7 years, CDC stage C 38.7%, HBV/HCV co-infection 25.7%. In pre-treated patients, 75.5% previously received NNRTIs (median duration on EFV and NVP of 480 and 396 days, respectively), 94.3% bPIs, 30.8% raltegravir (RAL) and 19.4% enfuvirtide. The most frequent ARVs associated with ETR were two NRTIs in 37.2% of the cases (21.9% in VF, 52.9% in MT), 1 bPI+RAL in 10.1% (13.5% in VF, 6.6% in MT), RAL in 6.2% (2% in VF, 10.5% in MT). Median duration on ETR was 3.7 and 2.2 years in the VF and MT group, respectively. In the VF group, HIV RNA was <50 c/ml in 71.7% (71.1% without bPI, 72% with bPI) of the patients at M12, 72.8% (71% without bPI, 73.3% with bPI) of the patients at M24. In the MT group, HIV RNA was<50 c/ml in 90.5% of the patients at M12 and 93.1% at M24. ETR was discontinued in 8.8% of the patients (12.8% in VF, 5.4% in MT) for adverse events in 23.9% of cases (21.5% in VF, 29.5% in MT), treatment failure in 15.2% (16.2% in VF, 7.4% in MT) or simplification in 5.4% (4.6% in VF, 7.4% in MT). In the VF group, factors associated with virologic failure in multivariate analysis were a longer duration of HIV infection (OR 2.6; 95% CI 1.7–4.0) and baseline HIV RNA >5 log10 c/ml (OR: 2.0; 95% CI 1.3–3.2) but not the association with a bPI.

**Conclusion:**

This large study shows that in ARV-pre-treated patients ETR is well tolerated with a high efficacy when combined with other active drugs, even when the regimen does not include a bPI.

